# Distracted Behavior of Pedestrians While Crossing Street: A Case Study in China

**DOI:** 10.3390/ijerph18010353

**Published:** 2021-01-05

**Authors:** Mingyu Hou, Jianchuan Cheng, Feng Xiao, Chenzhu Wang

**Affiliations:** School of Transportation, Southeast University, Nanjing 211189, China; 230179611@seu.edu.cn (M.H.); fengxiao@seu.edu.cn (F.X.); 230198272@seu.edu.cn (C.W.)

**Keywords:** mobile phone use, distracted behavior, theory of planned behavior (TPB), street crossing, pedestrian safety

## Abstract

Pedestrians are the most vulnerable road users in the traffic system and thousands of pedestrians are injured or killed globally as a result of traffic crashes every year. With their popularity and enriched functions, mobile phones are playing an increasingly important role in people’s lives, and records of vehicle crashes involving pedestrians have shown the hazards caused by distraction of mobile phone use, especially in the context of crossing the street. The present study employed the theory of planned behavior (TPB) to investigate the behavior of using a mobile phone while crossing the street in China. An online questionnaire based on the TPB framework was developed to collect data, and 387 eligible samples were retained after inspection. Mobile phone use while crossing the street is prevalent in China (i.e., 53%). The results show that three standard TPB constructs (i.e., attitudes, intention and perceived behavioral control) emerged as significant predictors of the behavior of using mobile phone while crossing, and two extended constructs (i.e., situation, mobile phone involvement) also significantly predicted the behavior. In addition, for this population, intention was the strongest predictor of the behavior among these significant constructs. Moreover, the results were discussed and compared with some existing studies and safety interventions were also provided.

## 1. Introduction

Traffic accidents that cause casualties are a major threat to public health worldwide. Thousands of pedestrians are killed in motor vehicle crashes each year. In China, for example, 15,123 pedestrians were killed and 31,683 were injured in 2015, representing 26.07% of the traffic fatalities and 15.85% of the traffic injuries, respectively [1]. Furthermore, in the past 10 years, the amount of pedestrians involving injuries and fatalities accounts for more than 30% of total casualties in motor vehicle crashes in urban areas [2]. In the U.S., it was reported 5987 pedestrians were killed in traffic crashes in 2016, which accounted for 16% of the total traffic fatalities [3]. These statistics all indicate the alarming situation of pedestrian safety. Before developing countermeasures, it is important to identify the factors that contribute to pedestrian-related crashes.

The majority of earlier studies on pedestrian safety have focused on the environmental factors (e.g., road width, signals) and driver-related factors; the contribution of pedestrians themselves were largely neglected [4,5]. In the past two decades, researchers have shown interests in personality traits and characteristics of pedestrians, especially the effect of demographic factors and unsafe behaviors on pedestrian safety [6]. For instance, Holland and Hill [7] found that compared to younger pedestrians, older people were less likely to intend to cross the streets in risky situations. Adolescent and middle-aged pedestrians were more likely to run a red light than older pedestrians [6,8]. Some studies have examined the gender difference in pedestrian behavior while crossing street, and female pedestrians were found less likely to run red lights [8,9,10].

As for the impact of unsafe behavior on traffic safety, more recently, researchers have paid increasing attention to distracted behavior among these vulnerable road users, in particular, the behavior involving mobile phone use while crossing street. A variety of portable devices, especially mobile phones, provide significant convenience and non-stop entertainment for users’ day-to-day life. According to statistical data from China Internet Network Information Center (CNNIC), the number of mobile netizens was 500 million and made up of 81% of the total number of netizens in 2013 [11], then the number increased to 753 million and 97.54% in 2017 [12]. Specifically, it is no exaggeration to say that the mobile phone has already become an indispensable device for Chinese people. In addition to basic usage like making phone calls and sending text messages, users can also go shopping, order food, call a taxi, or make a payment using the device at anytime. In a word, the mobile phone has changed the way people live.

Besides its convenience and benefits mentioned above, inappropriate use of the device may also cause negative effects to their users. Specific to traffic scenarios, it is well established that drivers who engage in mobile phone use while driving would experience a higher level of risk, and driving performance would be significantly compromised [13,14,15,16]. 

In recent years, researchers in the traffic safety community have expressed concerns about the potential risk resulting from the use of a mobile phone while crossing the street among pedestrians, and it is reported that some have paid the ultimate price [17]. Based on the emergency department data from National Electronic Injury Surveillance System (NEISS) in the U.S., Nasar and Troyer [18] estimated that 1506 pedestrians were injured in public places due to mobile phone use in 2010. Similar findings reported in previous studies provide more support for the negative effect of problematic device use on pedestrian safety. 

The evidences from some observational studies which focused on distracted behavior of pedestrians have confirmed that using a mobile phone while crossing the street would potentially increase crash risk [19,20,21]. Zhang et al. [22] found that pedestrians engaged in mobile phone use while crossing were more likely to be hit or almost hit by an oncoming vehicle at unsignalized intersections. Schwebel et al. [23] conducted an experiment to examine pedestrian behavior under four scenarios (i.e., distracted by phone conversation, texting, listening to music, and a no-distraction group) within an interactive and semi-immersive virtual environment; the results indicated that distraction caused by mobile phone use would reduce cognitive and visual capacity required to safely cross the street, and those distracted pedestrians looked away from street environment more often than the undistracted ones. In another study, Byington and Schwebel [24] adopted the same method of a semi-immersive virtual environment, but considered mobile internet use while crossing, and the findings again confirmed that pedestrian behavior was influenced and generally riskier. Moreover, distracted pedestrians waited longer and missed more safe opportunities to cross, as well as looked left and right less often than those do not use the device [22,23,24].

Given that mobile phones are increasingly popular among all age groups in everyday life, and considering their prevalent use in traffic scenarios (e.g., drivers, pedestrians), it is possible that the situation may even worsen in the future. In order to reduce pedestrian casualty due to distraction related to mobile phone use, it is fundamental to explore such unsafe behavior among pedestrians, along with their intention to cross under the condition of being distracted. Consequently, to better understand the causation and motivation behind such unsafe behavior, the theory of planned behavior (TPB) was employed as the theoretical framework in present study.

### 1.1. Theory of Planned Behavior (TPB)

The theory of planned behavior (TPB) is a well-validated model on decision-making and behavioral prediction. According to the TPB, intentions are summaries of people’s motivation to engage in an actual behavior [25], which in turn can be determined by attitudes towards the behavior, subjective norms (SN), and perceived behavioral control (PBC), and influences of the first two constructs can be moderated by the last one [26]. Attitudes towards behavior refer to individual’s positive or negative evaluation for the outcomes of a given behavior. SN is a degree of social pressure the individual perceives to perform the behavior, and the motivation to comply with those referent groups, such as parents and friends. PBC is a construct concerning how easy or difficult it is for the individual to perform a given behavior. Moreover, the construct of behavior is considered as a function of intentions and PBC in the model [27].

TPB has been widely utilized in predicting individual’s intentions and behavior, and its utility has been validated across a variety of research domains, including smoking [28] and drinking [29], etc. TPB has also been applied to investigate traffic safety involving human factors, such as driver’s intention and behavior. Elliott et al. [25] conducted a questionnaire study based on the TPB framework to identify belief predictors of TPB variables underpinning drivers’ intentions to comply with speed limits. Zhou et al. [30] found that TPB was able to explain 43% and 48% of the variance in hand-free and handheld mobile phone use intention while driving. Nemme and White [31] studied young people’s texting intentions and behavior while driving in Australia, including reading and sending text messages, they found that effects of standard constructs of TPB on intentions to send and read messages while driving were different. Attitudes emerged as significant predictors of intentions to both read and send texts while driving, and it was the strongest predictor. PBC and SN only significantly predicted the intentions to send texts while driving, but not the intentions to read texts. Furthermore, intention was the significant predictor for the behaviors of both sending and reading messages while driving, while PBC, attitude and SNs did not emerge as significant predictors for these two kinds of distracted driving behavior. 

Similarly, a number of existing studies have supported the validity of TPB in explaining pedestrians’ decision making while crossing [7,9,10,32]. Specific to pedestrians’ mobile phone use while crossing, Piazza et al. [33] surveyed 480 undergraduate students aged from 18 to 24 years using a questionnaire, the results showed that three constructs of TPB(attitudes toward the behavior, PBC, SN) significantly predicted the intention to use a mobile device while crossing street, attitudes emerged as the strongest predictor while PBC was the weakest one, and the model explained 48.4% of the variance in intention. It should be noted that distracted behavior involving mobile phone use in their study referred to: (1) send/view text messages; (2) view internet content; (3) mobile apps-related user behavior; (4) phone/video call. In other words, they did not differentiate the distraction type related to specific mobile phone usage in questionnaire design, such as view text messages (visual distraction), send text messages (visual and cognitive distraction). 

In another study, Jiang et al. [34] employed TPB to investigate a sample of 405 college students’ intention to use a mobile phone while crossing street. They also used a general questionnaire as well as constructs not differentiating aspects of distraction result from mobile phone use, and the device use was defined as: (1) make phone calls; (2) listen to music; (3) text messaging; (4) video entertainment. The results indicated that attitude, PBC and three extended constructs emerged as predictors of behavioral intention, while subjective norm was not of significant predictor. Three standard TPB constructs accounted for 13.3% of variance in intention when controlling for demographic variables, and attitude was the strongest predictor.

In a sample of 80 adults aged between 18 and 30, Barton et al. [4] examined the intentions to cross under four types of scenarios by using a questionnaire structured around TPB, the scenarios were: (1) texting; (2) listening to music; (3) receiving a phone call; (4) using applications. The differences within each TPB variable across the four scenarios were examined in their study. Finally, aggregate construct scores (i.e., not differentiating the scenarios, or aspects of distraction) were used to examine the predictors of behavioral intention. Three TPB variables totally accounted for 61% of variance in intentions, and PBC emerged as the strongest predictor, while SN was not a predictor with statistical significance. 

Lennon et al. [35] performed an online survey completed by 362 participants, and self-reported frequency of mobile phone use for three levels of distraction while crossing the street was obtained, including: (1) texting/internet accessing, namely visual and cognitive distraction; (2) voice calls, cognitive distraction; (3) listening to music—auditory only. It was found that 20% of the participants had high exposure to mobile phone use while crossing street. In order to control the duration of the questionnaire survey and completion time for participants, which may increase risk of biasing who might be willing to participant in the survey, only the distracting behavior of texting/internet accessing while crossing the street was investigated using a TPB-based questionnaire. For the entire sample (18–65 year olds), attitude and SN significantly predicted the intentions to cross the road while using a smart phone for text/internet access, and attitude was the strongest predictor, a total of 62% variance in intentions was explained by the three TPB variables. For 18–30-year-olds, all the TPB constructs were significant predictors of the intentions to cross the road while distracted by texting/internet accessing, accounting for 54% of the variance in such intentions, and attitude was suggested as the strongest predictor, while PBC was the weakest predictor.

### 1.2. The Present Study

Given that mobile phone use is rapidly increasing in this age of information, and using the device in pedestrian environment would place users at risk of being involved in traffic accidents, especially for those crossing the street, a number of previous studies examined association between psychological factors and mobile phone use among pedestrians on the basis of TPB. However, to the authors’ knowledge, very limited studies on this topic were carried out in a sample of Chinese population. In addition, previous studies demonstrated that traffic behavior might differ across countries due to the differences in culture, socio-economic factors and user habits [15]. Consequently, the utility of TPB for a Chinese sample is possibly different from the results reported in existing research conducted in other countries. Additionally, in most previous studies based on the TPB framework, the constructs were used to predict behavioral intention (i.e., intention was a dependent variable in regression model), not the behavior. However, although behavioral intention is considered as the antecedent of behavior, it does not mean that the behavioral intention will necessarily lead to the actual behavior. Thus, it is possible that taking the actual behavior as the dependent variable may be more useful in understanding specific behavior and its contributing factors. Finally, taken together, the aims of present study are to:

1. Validate the utility of TPB to predict actual behavior of using a mobile phone while crossing the street among pedestrians in China;
2. Examine the validity of three extended variables within TPB framework in predicting distracted behavior regarding mobile phone use while crossing street.
3. Illustrate the extent to which pedestrians’ distracted behavior is influenced by the constructs within TPB framework.

## 2. Methodology

### 2.1. Participants

This study was carried out with an on line survey in which a self-administered questionnaire was published on a Chinese professional survey platform—Sojump (www.sojump.com). A total of 420 participants responded to the online survey, and informed consents were obtained from all the participants. After the inspection of questionnaire results, 33 participants were excluded from the subsequent analysis due to their responses did not meet eligible criteria. The quality control strategy mainly included the time it takes to complete the questionnaire (in terms of a pilot survey and similar study experiences, at least 8 min is eligible in the present study) and consistency check of the responses. Thus, the final sample size was 387 participants from 10 provinces in China, consisting of 219 males (56.6%) and 168 females (43.4%). The age distribution is shown below: 17–18 years (*n* = 18, 4.7%), 19–25 years (*n* = 118, 30.5%), 26–30 years (*n* = 181, 46.8%), 31–40 years (*n* = 41, 10.6%), 41–50 years (*n* = 23, 5.9%), 51–60 years (*n* = 6, 1.6%). 247 participants (63.8%) hold a driver’s license. Table 1 presents the details of participants in this study.

### 2.2. Measures and Procedure

All respondents completed an anonymous questionnaire online, and researchers promoted the study through personal contacts, e-mails, and mobile social applications (e.g., QQ, WeChat) to get more participants. At the beginning of the questionnaire, participants would read an introduction to this survey, and were informed that personal privacy will be protected. If the respondent consented with the terms mentioned then the survey continued. Furthermore, after submitting the completed questionnaire, participants would get a red packet with cash in return.

In this study, the use of a mobile phone while crossing was defined as follows: (1) sending or reading text messages, browsing website pages, e-mailing via mobile phone; (2) watching videos; (3) listening to music; (4) talking on the mobile phone; (5) other activities involving mobile phone use. Additionally, the crossing scenario included intersection with and/or without signals.

#### 2.2.1. Demographic Measures

Demographic measures consisted of fifteen items, including gender, age, education background, whether a driver’s license holder, occupation, and other items in relation to daily mobile phone use history (e.g., total hours of mobile phone use per day, the type of usage that most hours spent in a day, the types/frequency of mobile phone use while crossing the street in the past). 

#### 2.2.2. TPB Questionnaire

The items measuring standard TPB constructs were created based on previous studies (e.g., [33,35]), and some items were appropriately modified according to objective of this study. All of the items were measured using a 5-point Likert scale ranging from 1 to 5. A higher score indicated participant’s higher tendency to cross when distracted by using mobile phone.

##### Behavioral Intention

Two items assessed respondent’s willingness and likelihood to cross road while using a mobile phone within the next two weeks, “it is likely that I will use a mobile phone for calling, texting, or other purposes while crossing the street (Item 1)” and “I intend to use a mobile phone for calling, texting, or other purposes while crossing the street (Item 2),” (1 = strongly disagree to 5 = strongly agree). Aggregate construct score of intention was determined by calculating the mean of item scores. The same approach was adopted for the other constructs. Moreover, internal consistency was examined by calculating Cronbach’s alpha, and it was reliable with α = 0.649. 

##### Attitudes

Four items were included to measure the construct of attitudes towards behavior. Fishbein and Ajzen [36] suggested that attitudes can be divided into two categories: “instrumental” and “experimental,” therefore, each aspect of attitudes was measured with 2 items in this study. For the instrumental aspect, “To use a mobile phone while crossing the street would make me keep informed with all valuable messages in real time, and it is important for me (Item 3),” and “To use a mobile phone while crossing the street allowed me to make full use of the time and deal with some important tasks (Item 4)” were used. The experimental aspects were about their emotional evaluation of the behavior, such as “For me, to use a mobile phone while crossing would be good/enjoyable (Items 5 and 6).” (Cronbach’s α = 0.839).

##### Subjective Norm (SN)

SN was assessed using a composite scale consisting of 2 items, “My parents and friends would think that I should use a mobile phone while crossing the street (Item 7),” and “I would like to cross street in the way my parents and friends think I should (Item 8).” Items were scored 1 (strongly disagree) to 5 (strongly agree), and a higher score indicated a favorable norm toward the behavior. (Cronbach’s α = 0.638).

##### Perceived Behavioral Control (PBC)

Four items measured construct of PBC, including “I have complete control over whether I would use a mobile phone while crossing the street (Item 9),” and “It would be easy for me to engage in the behavior of using a mobile phone while crossing the street (Item 10),” “Whether or not to use a mobile phone while crossing the street just depends on me (Item 11),” “I am confident that I can use a mobile phone while crossing the street (Item 12).” Similarly, the mean of four items was produced as the overall measure of PBC. (Cronbach’s α = 0.838)

#### 2.2.3. Extended Measures

##### Mobile Phone Involvement (MPI)

It was presumed that distracted behavior the present study focused on maybe impacted by the mobile phone involvement or addiction factors [35], namely that using the device while crossing was a specific scenario which may reflect the user’s daily use habits to a certain extent. MPI was assessed with three items measuring the addiction to mobile phone. These three items were: (i) “Being without my mobile phone makes me feel distressed (Item 13);” (ii) “It is hard for me to reduce the time spending on mobile phone use (Item 14);” (iii) “I think that I am addicted to using mobile phone in daily life (Item 15).” Similarly, a higher score meant stronger dependence on mobile phone. (Cronbach’s α = 0.849).

##### Safety Awareness

Two items were proposed to measure the influence of safety awareness on occurrence of distracted behavior: “I think to cross street while using a mobile phone would be very unsafe (Item 16),” and “When I cross together with my friends, if they were using mobile phones while crossing street, then I will stop them (Item 17).” Before calculating the mean of these item scores, reverse-coding was carried out, so that a higher score indicates a weaker safety awareness, and higher likelihood to perform the distracted behavior. (Cronbach’s α = 0.686). 

##### Situation

The construct of situation was assessed with two items mainly measuring the influence of other pedestrians’ behavior of using a mobile phone (i.e., conformity tendency) and considering about the scenario of crossing alone (i.e., without a companion). The two items were as follows: “When crossing the street, if the other pedestrians around me are using a mobile phone, so I also want to use my phone at that moment (Item 18),” and “When I cross the street alone, I feel bored so that I would like to use my mobile phone while crossing (Item 19).” Higher scores indicated greater intention to cross street while using mobile phones. These two responses were averaged to present the overall measure of situation. (Cronbach’s α = 0.714).

### 2.3. Analyses

Binary logistic regression analyses were conducted to examine the utility of aforementioned constructs within TPB framework for predicting the behavior (i.e., previous behavior of using a mobile phone while crossing). According to data property, Chi-Square tests were performed to compare the differences in demographic measures mainly grouped by age and gender. Moreover, a series of MANOVA, one-way ANOVA and nonparametric tests were used to investigate the differences between construct means/medians grouped by the demographic variables. Meanwhile, the descriptive statistics were computed as well.

## 3. Results

### 3.1. Descriptive Analysis

Table 2 provides the descriptive statistics based on age and gender groups, including the frequency and percentage of corresponding population. The results of Chi-Square tests were also presented in the Table 2. Regarding daily mobile phone use, 75 male (34.2%) and 58 female (34.5%) participants responded they spend about 2–4 h per day on mobile phone use which were groups with the largest proportions in each gender population, followed by 4–6 h (32.4% and 32.7%, respectively). According to the Chi-Square test, these two factors were independent of each other. That is to say the difference in daily mobile phone use between males and females was not statistically significant (*p* > 0.05). Indeed, the proportions for males and females were quite comparable within each pairs. In contrast to gender, the daily mobile phone use among age groups were somewhat different (*p* < 0.05): compared to the younger participants, the older ones spend much less time on device use. In other words, the result showed that young adults were the population of the most frequent users of mobile phones. Thus, to some extent, distracted crossing was more likely to happen among young adults.

Reported main usage of mobile phone on a daily basis was similar between males and females (*p* > 0.05). Participants spent the most time on social applications. Indeed, a variety of social apps are prevalent in China due to their entertainment and communication functions, such as Wechat, QQ and Sina Microblog. Among the age groups, the percentage for younger participants on social apps option was greater than the older, 41.4% of 41–60 year olds chose “others”, and none of these participants chose “play games” category. 

As for whether they had experience of using mobile phones while crossing in past two weeks, over half of males (50.7%) and females (56.5%) reported “Yes.” Although the proportion for females to do so was somewhat higher, but no statistically significant difference was found (*p* > 0.05). In contrast, differences emerged across the age groups on previous experience (*p* < 0.05): the younger participants were more likely to engage in the distracted behavior, while 21 (72.4%) of over 41 year olds did not have such experience.

In addition, this study also considered the main usage of device during street crossing among pedestrians in China, the most frequently chosen option for male (49.5%) and female (54.7%) participants was phone calls, followed by social apps (23.4% and 31.6%). One previous study conducted by Byington and Schwebel [24] found that the most common reason for pedestrians using mobile internet while crossing was related to the use of social apps (23.9% of 92 participants). Just from this point of view (i.e., mobile phone usage involving internet), the present study was consistent with the previous one. Furthermore, females were less likely to be distracted by playing games than males. The result of Chi-Square test showed that the difference across age groups was statistically significant (*p* < 0.05). Specifically, over half of participants in group 31–40 years and over 41years reported that they used the device for voice calls (63.2% and 75.0%) while crossing street, followed by social apps related usage (26.3% and 12.5%). It seems that most respondents who using mobile phones while crossing due to communication-related reasons rather than entertainment-related requirements.

### 3.2. Differences in Constructs

Before comparing the differences in constructs and using a binary logistic regression model to predict the past behavior (i.e., the behavior of using a mobile phone while crossing the street before), factor extraction was conducted. First, an exploratory factor analysis (EFA) was performed to identify the construct validity of 7 components in present study (KMO = 0.895, Bartlett’s test *p* < 0.001). A principal component analysis with fixed number of factors was applied to reduce dimensions of all 19 items, then 7 identified factors which were consistent with the factors previously defined within TPB framework accounted for a total of 75.18% of the variance. In addition, the varimax rotation method was used to display factor score coefficient matrix and used these factors as 7 independents (i.e., 4 standard and 3 extended constructs) for logistic regression analysis. Table 3 presents the details of factor loadings.

All aggregate measures were compared between males and females, respectively. Due to the violation of homogeneity of variance, two methods were used here, MANOVA (including attitudes, intention, MPI and situation) and *t*-test (including SN, PBC and safety awareness). The results of MANOVA analysis indicated that no significant multivariate main effect was found for gender, with *F*(4, 382) = 0.868, *p* > 0.05, Wilk’s Lambda = 0.991. On the other hand, by using *t*-test, significant difference in SN and safety awareness was found, but not in construct PBC. The result suggested that males had higher scores than females on both constructs (i.e., 1.566 vs. 1.417, 2.160 vs. 1.854). Consequently, it could be concluded that males perceived a slightly greater favorable norm compared to their counterparts, at the same time, they were also more sensitive to traffic risk results from distracted crossing behavior. Such phenomenon may be attributed to the role of males and females in mundane life. The differences in constructs between participant groups were detailed in Table 4.

Similar to gender, difference in constructs among daily mobile phone use groups was examined. In terms of MANOVA results (including SN, PBC, intention and MPI), a significant multivariate main effect was found, *F*(12, 1006) = 4.709, *p* < 0.05, Wilk’s Lambda = 0.865. Moreover, significant univariate main effects were found for intention, *F*(3, 383) = 8.415, *p* < 0.001, *η*^2^ = 0.062, and MPI, *F*(3, 383) = 14.288, *p* < 0.001, *η*^2^ = 0.101. Subsequently, pairwise comparisons were conducted for the two constructs respectively, and the results were similar: those participants who spend more hours on mobile phone use per day had higher scores than the participants who spend less time on device use, namely participants in the first group had the smallest score, while participants in the last group had the highest score (Intention: 1.780 vs. 2.806; MPI: 2.240 vs. 3.204). Table 4 presents the details of comparisons as well as mean values for each group. And it was as expected that the more time spent on device use, the more addicted the person was, finally a higher responded score for MPI would be obtained. Consequently, it could be reasonably inferred that for the individual who spent more time on device use, she/he was also more likely to engage in such behavior while crossing street. In other words, the intention to use mobile phone while crossing was influenced by their daily use habits, these two variables were positively correlated. There were no significant differences in PBC/SN between the groups (*p* > 0.05).

The other three constructs were investigated by Kruskal–Wallis analyses, and the results indicated significant differences among groups within situation and attitude (*p* < 0.05), but not for safety awareness (*p* > 0.05). In order to further study the mean difference between groups, post-hoc tests with a Bonferroni correction (i.e., ANOVA) were performed. For pairwise comparisons on attitudes, the only difference between the first and last group was significant (*p* < 0.05). Means, medians and mean ranks were shown below: *T* ≤ 2 (mean = 1.390, median = 1.00, mean rank = 128.02), 6 < *T* (mean = 1.862, median = 1.75, mean rank = 211.32). Results of pairwise comparisons for construct situation were similar to attitudes, participants in the first group had a significant lower score than those in the last group: *T* ≤ 2 (mean = 1.500, median = 1.00, mean rank = 133.18), 6 < *T* (mean = 2.078, median = 2.00, mean rank = 204.47). As a whole, it was found that the individuals who spent more hours on mobile phone-related activities daily also had a more positive attitudes toward the using behavior when crossing street, and their behavior of using the device were also more likely to be influenced by others and/or surrounding.

For the variable of previous exposure to pedestrian injury/critical event, 35.66% (138) of participants responded that they had either bumped into other pedestrians or stationary objects (e.g., trash cans/lamp posts), and even had some more dangerous experiences (e.g., a close call with a vehicle) that directly caused by distraction of using mobile phone while walking. The remaining 249 participants (64.34%) responded no exposure. The difference between the two groups was examined by a series of *t*-tests. The results revealed that there were significant differences in all constructs between the two groups (*p* < 0.05), the details were presented in Table 4. Meanwhile, it should be noted that the pattern for each construct was similar: the group in which participants reported such an exposure had a higher score than the group that reported no exposure. Specifically, compared with those not reporting exposure to pedestrian injury/critical event, participants who reported such an exposure perceived more support from significant others (1.652 vs. 1.418), valued the behavior in a more positive way (1.908 vs. 1.605), and showed more confidence in the ability of behavioral control (1.877 vs. 1.605) and their behavioral intention to use a mobile phone in a street crossing scenario was significantly higher than their counterparts (2.757 vs. 2.361). In addition, it was also found that the participants in exposure group were more addicted to the device use than those in non-exposure group (3.044 vs. 2.819), and their safety awareness was relatively weak (2.196 vs. 1.934). As a result, it was not surprising that they were more likely to be involved in pedestrian injury/critical event. It seems that there may be potential relationships between the previous exposure and these psychological constructs.

A series of t-tests were carried out to examine the difference in constructs between participants who reported using a mobile phone while crossing and not using the device. The results demonstrated that there were significant differences between the two groups for all the constructs. Compared with the participants who did not have an experience of using mobile phone while crossing the street in the past, those distracted pedestrians had significant higher scores on constructs (see in Table 4), indicating that they had more positive attitudes towards such distracting behavior (2.027 vs. 1.356), perceived higher control over the behavior (1.975 vs. 1.392), had weaker safety awareness (2.180 vs. 1.854), were also more addicted to mobile phone use in their daily life (3.071 VS 2.704) and had higher intention to perform the behavior (2.932 vs. 2.014).Moreover, the participants reporting a past experience of distracted crossing were significantly more susceptible to the situation factor than those not reporting such an experience (2.296 vs. 1.588), and the former group also scored higher on construct SN (1.653 vs. 1.329). Consequently, they were indeed more likely to use a mobile phone during street crossing, and they also did so. To some extent, the results indicated that there may be potential associations between these constructs and behavior.

In order to examine the difference in constructs between age groups, MANOVA were performed for intention, MPI and situation, a significant multivariate main effect was found, *F*(12, 1006) = 4.165, *p* < 0.05, Wilk’s Lambda = 0.880. Furthermore, the results showed that there were univariate main effects for intention *F*(4, 382) = 5.157, *p* < 0.001, *η*^2^ = 0.051, and MPI, *F*(4, 382) = 6.186, *p* < 0.001, *η*^2^ = 0.061, the age difference in construct situation was not significant (*p* > 0.05). Additional pairwise comparisons were conducted for intention and MPI (please see Table 4 for details). With regards to intention, it was found that the youngest participants reported a higher level of intention to perform the distracted behavior than the oldest group (*p* < 0.05, 2.917 vs. 1.931), indicating that the younger participants were more likely to be distracted. Moreover, significant age differences were also found between 26–30 year olds and 41–60 year olds (*p* < 0.01, 2.649 vs. 1.931). The results of other pairwise comparisons were not statistically significant. With regards to MPI, participants aged 19–25, 26–30 and 31–40 years were significantly more addicted to mobile phone use than those over 41 years old (*p* < 0.05, 2.794/2.996/3.195 vs. 2.264). This phenomenon may be not surprising given that younger people are more susceptible to the new technologies and electronic devices than the older ones in general.

The age differences in SN, PBC, SA and attitudes were examined using Kruskal–Wallis analyses due to the violations of homogeneity of variance. The significant differences between age groups were found for PBC and attitudes (*p* < 0.01), while the differences in SN and SA were not statistically significant (*p* > 0.05). With respect to PBC, it was found that the younger participants had significantly higher scores than older participants (*p* < 0.01). The results of post-hoc tests with Bonferroni correction found that 17–18-year-olds had significantly higher scores than 31–40 as well as 41–60 year olds (*p* < 0.01, 2.167 vs. 1.512/1.336), while the remaining pairwise comparisons were not significant (*p* > 0.05). These results indicated that the older individuals’ perception of their ability to perform the distracted crossing involving mobile phone use was lower than the younger individuals’, this may be largely due to the difference in physical function between ages, such as the hearing of older people may be less good, and they respond slowly to a sudden event in general. As for attitudes, the results indicated that younger participants had higher scores than the older participants (*p* < 0.01), indicating that younger participants had a more positive attitude towards the behavior. Furthermore, according to the results of post-hoc tests with Bonferroni correction, the differences between age groups 17–18 and 41–60 (*p* < 0.01, 2.097 vs. 1.345), and group 19–25 and 41–60 (*p* < 0.05, 1.788 vs. 1.345) were found, the remaining pairwise comparisons were not statistically significant (*p* > 0.05).

### 3.3. Correlations, Predictors of the Behavior

Spearman correlations, means and standard deviations for all of the constructs were presented in Table 5. Except for the construct of intention and MPI, means for the other components were below midpoint scale (5-point Likert scale), which indicated that pedestrians had a relatively negative attitudes towards the distracted behavior in present sample. All constructs were significantly correlated with past behavior (*p* < 0.001), including standard and extended TPB constructs. Specifically, the strongest correlation was found between behavior and attitudes, followed by behavior and intention, while it had the weakest correlation with MPI. Moreover, most constructs were positively correlated with each other, only a negative relationship was found for safety awareness and MPI, but it was weak and not statistically significant. It was found that the relationships between standard TPB constructs were strong, with most the correlation coefficients were higher than 0.4. In particular, the correlation between PBC and attitudes was the strongest with a coefficient of 0.698, indicating that the participant who perceives that he/she is more capable of performing the behavior may also have more positive attitudes towards such behavior, as a result, he/she is more likely to perform the behavior, namely to use a mobile phone while crossing the street in the current study. 

Finally, past behavior (i.e., a binary dependent variable) was regressed on the seven components based on TPB framework and five demographic variables in a binary logistic model. The results were shown in Table 6. Safety awareness, subjective norm and five demographic variables, including gender, age, education background, daily mobile phone use and whether a driver’s license holder, did not emerge as significant predictors in predicting the behavior. The remaining five constructs with statistical significance positively related to actual behavior of using a mobile phone while crossing street, and intention was the strongest predictor while MPI was the weakest one. Specifically, of three standard TPB constructs, behavioral intention was found to be the strongest component to predict behavior while PBC was the weakest predictor.

## 4. Discussion

### 4.1. Predictive Efficacy of the TPB

The present study applied TPB to investigate the behavior of crossing the street while under the distracted condition of using a mobile phone in a Chinese population. In addition, a number of demographic measures related to device use in daily as well as while crossing were examined. Correlations between constructs were analyzed as well. Findings validated the utility of TPB in predicting the distracted behavior involving mobile phone use while crossing the street among pedestrians in China.

In the present study, a total of five constructs emerged as significant predictors for the actual behavior, including three standard TPB constructs (intention, attitudes, PBC) and two extended constructs (situation, MPI). Among these significant predictors, intention was the strongest one.

For standard TPB constructs, behavioral intention, not PBC, was the strongest predictor of behavior. This result was similar to that of a study conducted by Nemme and White [31], which found intention to be the only predictor with statistical significance among standard TPB constructs to predict behavior of sending and reading texts while driving, and PBC did not even meet the significance requirement. Another study by Walsh et al. [37] suggested the fact that PBC did not significantly predict behavior may be more reflective of the volitional nature of mobile phone use, because it is a prevalent behavior among pedestrians. The positive relationship between intention and behavior indicated that pedestrians with higher tendency and intent to cross street while using mobile phones would be more likely to do so.

The findings for attitudes were contrary to the research conducted by Nemme and White [31], they argued that attitudes could not significantly predict behavior of sending and reading texts while driving. However, in this study, attitudes was a more significant construct than PBC to predict the behavior, and it was comparable to intention which was the strongest predictor. Similarly, as found by Ledesma et al. [38], both implicit attitudes and explicit attitudes made a significant contribution to the explanation of seatbelt use, and the former one had a direct effect on seatbelt use in terms of path analysis. When considering behavioral intention as the dependent variable, attitudes emerged as the strongest predictor in a number of previous studies [31,33,35], while as the weakest one in [30]. Furthermore, Zhou, Horrey and Yu [9] found that attitudes was the strongest predictor of pedestrians’ intentions to cross against a traffic signal under the non-conformity situation, but it was the weakest one under conformity situation. In another of their study [32], the findings were also similar. As for the present study, it was found that the individual with a positive attitudes was more likely to perform the distracted behavior.

With regard to present sample, PBC emerged as a significant predictor of behavior, while Nemme and White [31] stated it did not reach the statistical significance level. Consistent with this study, Ledesma et al. [38] indicated that PBC significantly predicted drivers’ behavior of seatbelt use when driving a vehicle. As for the effect of PBC in predicting intention, Piazza et al. [33] and Lennon et al. [35] found PBC was the weakest predictor while Barton et al. [4] suggested it was the strongest one. Additionally, Nemme and White [31] found PBC emerged as the second strongest predictor of intention to send texts while driving, but with no statistical significance for reading. Results of another study in driving safety field indicated that PBC was the strongest predictor of intention to use either a handheld or hands-free mobile phone while driving [30]. 

According to the result of regression model, SN was not a construct which could significantly predict the behavior. Similarly, Nemme and White [31] found that SN neither significantly predict the behavior of sending nor reading texts while driving. Moreover, SN was a significant predictor for intention to send texts while driving, but not to read texts. In some other studies which investigated predictor of pedestrian intention, the findings also varied across studies. For instance, Piazza et al. [33] examined the intention of mobile phone use while crossing with a sample of 480 undergraduate students, and SN emerged as a significant predictor in their multiple regression model. Similar results were reported in [32]. A total of 80 undergraduates aged 18 to 30 were recruited in the study of [4], it was found that SN could not significantly predict pedestrians’ intention to cross while being distracted by mobile phone. For this study, a positive relationship between SN and distracted behavior implied that pedestrians who perceived a higher level of support for such behavior from important others (e.g., parents, friends) were also more likely to do so.

For three extended constructs within TPB framework, evidence was found for the ability of situation and MPI to predict actual behavior. Specifically, situation was a predictor even stronger than PBC, while MPI was the weakest one among these five significant predictors. Similarly, Lennon et al. [35] found that MPI could significantly and positively predicted behavioral intention to use smart phone while crossing the street in a sample of 362 participants. Furthermore, in their study, according to beta weight for each variable obtained from regression model, the effect of MPI on intention was greater than construct PBC, and was comparable to SN, but less than attitudes. This positive relationship indicated that pedestrians who spend more time on daily mobile phone use (i.e., they were more addicted to device use) were also more likely to use the device when participating in traffic activities, such as crossing street. In fact, the finding concerning MPI was as expected, the authors assumed that mobile phone use while crossing was neither a completely random behavior nor an indispensable action under such traffic scenario, this type of distracted behavior may relate to individual’s daily use habits, especially the extent that individuals addicted to the mobile device use may influence its use while crossing. 

The findings for situation demonstrated that some situational factors may potentially affect road users’ behavior. As for the conformity facet within situation, it was considered in previous studies about pedestrians’ behavior of jaywalking [32], but to the authors’ knowledge, this was the first time that conformity was considered as a factor in investigating the distracted behavior related to mobile phone use while crossing street. In future research, perhaps conformity should be included as an independent construct within TPB framework, and further research should be conducted to examine its effect. Additionally, walking without a companion was another facet of situation considered in this study, it was demonstrated that the respondent was more likely to use device when he/she was walking alone. Probably, it was boring, particularly for younger pedestrians who were travelling alone, then at such a moment, the smart phone was no longer just a device, but also a companion who could make the travel interesting, at least not so boring. 

At the beginning of data analysis, the authors assumed that pedestrians who use mobile phones while crossing were insensitive to the risk result from distraction, namely they had weak safety awareness. This assumption was partly confirmed in terms of a positive association between safety awareness and past behavior (see in Table 5). However, safety awareness did not emerge as a significant predictor in current study. Additionally, the score (mean = 2.027) indicated that most respondents thought using mobile phone while crossing the street was dangerous. A possible explanation for inconsistency between safety awareness and actual behavior maybe that although pedestrians perceived that crossing the street while being distracted was unsafe, but it was really common to use the device in contemporary secular life, and even to use it while crossing may not be a challenge for most people. Moreover, given that pedestrian accident was an event with small probability, the risk due to mobile phone use was acceptable for most pedestrians. Hence, it was not surprising that pedestrians may engage in mobile phone use while crossing despite their regard that such behavior is unsafe. 

Taken together, the utility of both standard and extended TPB constructs in understanding along with predicting pedestrian behavior of using mobile phone while crossing the street were validated. Moreover, although it was found that the relative contributions of constructs in explaining behavior or intention were different among studies, this phenomenon was not surprising, given that the specific contributions and statistical significance of constructs depend on the factors such as sample characteristics and research scenarios [31,33,36,37].

### 4.2. Implications for Practice

Compared to just simply testing the utility of TPB in traffic scenarios, developing safety countermeasures using TPB is more important and valuable. In terms of the findings in present study, it seems feasible to change constructs within the TPB framework and ultimately affect on the intention and behavior. In other words, pedestrian safety could be improved by self-consideration—of the pedestrians’ role themselves, as opposed to drivers or traffic properties. 

For the present study, MPI emerged as a significant predictor of behavior, suggesting that the behavior of using a mobile phone while crossing is associated with users’ daily habits. To some extent, heavy device users are more likely to perform this type of behavior, and maybe it is just habitual or subconscious behavior to use it in a traffic scenario. From this point of view, education campaigns targeting pedestrians are necessary to make pedestrians pay more attention to distracted behavior concerning mobile phone use while street crossing and the associated hazards, especially for those younger populations who are frequent users of mobile phones. Moreover, all should be aware of how to appropriately use mobile phones (e.g., the time and place you use it), in particular, when multitasking.

For construct of situation, the concept that conformity tendency to use mobile phone in such a situation will lead to negative results should be promoted among pedestrians, especially the individuals who have a higher tendency to conform others in day-to-day life. People intending to use a mobile phone while crossing due to walking alone or just want of stimulation should keep in mind that “safety is always more important than entertainment, pedestrians should focus on the way and traffic”.

Attitude significantly predicts the behavior in the logistic model, and a positive association between attitudes and behavior as well as intention is found according to correlation analysis. Hence, efforts to change positive attitudes towards inappropriate device use (e.g., it is valuable) may be useful in preventing distraction among pedestrians. Messages that highlight the risk of distracted behavior while crossing should be delivered. In addition, road safety education to emphasize the disadvantages of using mobile devices while street crossing would be effective as well. More importantly, to cultivate positive attitudes towards road safety among younger road users has a profound impact on individuals’ long term road safety outcomes concerning their health and welfare [13,39]. 

Despite that SN is not a significant predictor of the behavior in this study, it positively correlates with both behavior and behavioral intention. Thus, interventions aims at SN may still work. As a part of public safety education or media awareness campaigns, interventions are achievable by promoting the message that important others (i.e., parents, friends, peers) do not approve the behavior of using mobile phone while crossing. In particular, prioritizing focus on SNs in teenager and university student populations may be more efficacious, given that they are heavy device users as well as are more desirable for social approval.

In addition, prevention strategies based on the other constructs (e.g., PBC) are also worthwhile. At the same time, it is worth mentioning that in addition to the safety interventions in relation to psychological factors mentioned above, to build pedestrian overpass or underpass at intersections with heavy pedestrians is a good choice to protect these vulnerable road users, no matter whether he/she is easily being distracted by mobile devices or not.

### 4.3. Limitations and Future Research

There are several limitations that must be considered when interpreting the results of this study. First, to examine the utility of the TPB in explaining pedestrians’ behavior or intention related to mobile phone use while crossing street, a self-reported questionnaire is proposed to collect data. Although a brief introduction was included to state background and purpose of the survey, and the anonymity of questionnaire was ensured, however, it was still hard to collect completely reliable responses due to social desirability bias. In this sense, some participants preferred to provide the “right answer”, but not the “real answer”, although such distracted behavior was not prohibited by laws and regulations in China, and the “no-existence of wrong or right response” was emphasized in the introduction section.

Second, the present study did not differentiate the type of mobile phone use while crossing (e.g., text messaging and phone calling), we just combined the various common device usages together. However, the scores of constructs within TPB framework are likely to differ across mobile phone usages. The future research should focus on specific type of mobile phone use, then to examine the utility of TPB in explaining pedestrian behavior. 

Third, the present study mainly focused on relationship between the distracted behavior and psychological factors. Given that the data is collected through an anonymous online survey, it is unlikely to collect subsequent data regarding distracted behavior of participants by a follow-up survey. On the other hand, in terms of some literature (e.g., [31]), “past behavior” is positively correlated with “behavior”. Consequently, “past behavior” was used to instead of “behavior” in the data analysis of present study. Such limitation should be noted when interpreting our results, and future studies are encouraged to conduct a follow-up survey. In addition, advanced tools mentioned in a previous study [33] may be helpful in implementing surveys in the future.

Fourth, the participants aged 19–30 years made up a large proportion (totally 77.3%) in the present sample, and it is possible that the perception of psychometric constructs differs across age groups, such as attitudes and PBC in the TPB framework. Thus, the difference in age distribution between this study and past studies may also contribute to their divergences in results. Additionally, the future studies are encouraged to use the samples with a more balanced age distribution. 

Finally, this study used a brief questionnaire which was developed based on TPB framework to explore distracted behavior among pedestrians, the number of items for the constructs ranged from 2 to 4. In order to improve the reliance of collected data as well as to investigate other contributing factors of such pedestrian behavior, the future studies should attempt to expand the number of items and develop more constructs.

## 5. Conclusions

The present study provides support for the utility of TPB in predicting the behavior of using mobile phone while crossing the street among pedestrians in China. In the sample of 387 participants with a wide age distribution, over half of the males and females had the experience of using a mobile phone while crossing, and most of these participants responded that the main purpose of device use was related to phone calls and social apps. Moreover, there were significant differences in mobile phone use and scores of constructs among cohorts. Finally, three of the four standard TPB constructs (i.e., attitudes, intention and PBC) and two of the three extended constructs (i.e., situation, MPI) emerged as significant predictors of the behavior in a binary logistic model, these constructs should be prioritized when developing safety interventions and policies. To some extent, the present study also contributes to the literature on utility of TPB framework in predicting behavior, rather than behavioral intention.

## Figures and Tables

**Table 1:** Number and percentage of participants by demographic variables (*N* = 387).

	*n*	Percent (%)		*n*	Percent (%)
**Gender**			**Age**		
Male	219	56.6	17–18	18	4.7
Female	168	43.4	19–25	118	30.5
**Driving license**			26–30	181	46.8
With	247	63.8	31–40	41	10.6
Without	140	36.2	41–50	23	5.9
**Occupation**			51–60	6	1.6
Student	109	28.2	**Education**		
Enterprise staff	143	37.0	Primary/Middle school	30	7.8
Government employee	38	9.8	High school	38	9.8
Self-employed	20	5.2	Undergraduate	251	64.9
Others	77	19.9	Postgraduate	68	17.6

**Table 2:** Participant demographic characteristics and Chi-Square Test.

	Gender (%)		*p*-Value	Age (%)					*p*-Value
**DMPU**	**Male**	**Female**	n.s.	**17–18**	**19–25**	**26–30**	**31–40**	**41–60**	<0.001
*T* ≤ 2	5.9	7.1		16.7	1.7	3.3	4.9	41.4	
2 < *T* ≤ 4	34.2	34.5		16.7	26.3	37.6	53.7	31.0	
4 < *T* ≤ 6	32.4	32.7		44.4	37.3	32.6	29.3	10.3	
6 < *T*	27.4	25.6		22.2	34.7	26.5	12.2	17.2	
**MUD**			n.s.						<0.001
Videos	18.3	22.0		16.7	22.9	20.4	14.6	13.8	
Social apps	49.3	56.5		50.0	58.5	54.7	41.5	31.0	
Games	5.5	2.4		16.7	5.9	2.8	2.4	0	
Novels/Web pages	10.0	7.1		5.6	4.2	9.9	14.6	13.8	
Others	16.9	11.9		11.1	8.5	12.2	26.8	41.4	
**PUMPC**			n.s.						0.016
Yes ^a^	50.7	56.5		61.1	50.8	59.7	46.3	27.6	
No ^b^	49.3	43.5		38.9	49.2	40.3	53.7	72.4	
**MUC**			0.047						0.023
Voice calls	49.5	54.7		36.4	41.7	55.6	63.2	75.0	
Videos	2.7	0		0	3.3	0	5.3	0	
Social apps	23.4	31.6		18.2	30.0	27.8	26.3	12.5	
Games	5.4	0		18.2	5.0	0.9	0	0	
Music	8.1	9.5		18.2	8.3	10.2	0	0	
Novels/Web pages	0.9	1.1		0	1.7	0	0	12.5	
Others	9.9	3.2		9.1	10.0	5.6	5.3	0	

Note: DMPU = daily mobile phone use (hours/day); MUD = main usage in daily; PUMPC = previous use of mobile phone while crossing; MUC = main usage while crossing; a = the response to past behavior of using mobile phone while crossing, including scores from 2 (sometimes) to 5 (always); b = the response to past behavior of using mobile phone while crossing with the score of 1 (i.e., never).

**Table 3:** Principal component analysis and Cronbachs.

	Factor						
Item	PBC	MPI-	Situation	Attitudes	SN	Intention	SA
1						0.860	
2						0.690	
3				0.760			
4				0.527			
5				0.460			
6				0.606			
7					0.773		
8					0.750		
9	0.694						
10	0.797						
11	0.804						
12	0.593						
13		0.901					
14		0.882					
15		0.807					
16							0.879
17							0.765
18			0.831				
19			0.711				
Cronbach’s *a*	0.838	0.849	0.714	0.839	0.638	0.649	0.686

Note: SN = subjective norm; PBC = perceived behavioral control; Intention = behavioral intention; MPI = mobile phone involvement; SA = safety awareness. Factor loadings under 0.40 were suppressed.

**Table 4:** Differences in constructs (mean) between participants grouped by demographic variables.

	SN	PBC	Attitudes	Intention	MPI	SA	Situation
**Gender**							
Male	1.566	1.759	1.712	2.555	2.869	2.160	1.952
Female	1.417	1.628	1.714	2.435	2.938	1.854	1.982
*p*-value	0.039	n.s.	n.s.	n.s.	n.s.	0.003	n.s.
**DMPU**							
*T* ≤ 2	1.420	1.400	1.390	1.780	2.240	2.060	1.500
2 < *T* ≤ 4	1.523	1.673	1.705	2.376	2.659	2.075	1.955
4 < *T* ≤ 6	1.512	1.734	1.665	2.532	3.034	2.012	1.976
6 < *T*	1.481	1.774	1.862	2.806	3.204	1.976	2.078
*p*-value	n.s.	n.s.	0.020	<0.001	<0.001	n.s.	0.039
**Exposure**							
Yes	1.652	1.877	1.908	2.757	3.044	2.196	2.196
No	1.418	1.605	1.605	2.361	2.819	1.934	1.837
*p*-value	0.003	0.002	<0.001	<0.001	0.016	0.018	<0.001
**PUMPC**							
Yes	1.653	1.975	2.027	2.932	3.071	2.180	2.296
No	1.329	1.392	1.356	2.014	2.704	1.854	1.588
*p*-value	<0.001	<0.001	<0.001	<0.001	<0.001	0.002	<0.001
**Age**							
17–18	1.611	2.167	2.097	2.917	2.963	2.083	2.361
19–25	1.610	1.754	1.788	2.462	2.794	2.212	2.038
26–30	1.497	1.724	1.709	2.649	2.996	1.961	1.925
31–40	1.292	1.512	1.610	2.195	3.195	1.854	2.012
41–60	1.310	1.336	1.345	1.931	2.264	1.897	1.603
*p*-value	n.s.	0.003	0.005	<0.001	<0.001	n.s.	n.s.

Note: Exposure = previous exposure to pedestrian injury/critical event; n.s. refers to *p* > 0.05; *p*-value refers to statistical significance level for the results of comparison.

**Table 5:** Spearman correlations between constructs.

Variable	2	3	4	5	6	7	8	*Mean*	*SD*
1. SN	0.588 ***	0.630 ***	0.363 ***	0.307 ***	0.063	0.464 ***	0.472 ***	1.501	0.727
2. PBC		0.698 ***	0.429 ***	0.437 ***	0.135 **	0.490 ***	0.549 ***	1.702	0.775
3. Attitudes			0.446 ***	0.507 ***	0.153 ***	0.410 ***	0.610 ***	1.713	0.728
4. Intention				0.474 ***	0.284 ***	0.327 ***	0.482 ***	2.502	1.019
5. Behavior					0.205 ***	0.242 ***	0.414 ***	1.600	0.654
6. MPI						−0.016	0.186 ***	2.899	0.884
7. SA							0.349 **	2.027	1.019
8. Situation								1.965	0.898

Note: Behavior = past behavior; *** *p* < 0.001, ** *p* < 0.01.

**Table 6:** Binary logistic regression model predicting the behavior.

Variable	B	S.E.	Wals	Exp(B)
Constant	0.268	0.885	0.092	1.308
PBC	0.646 ***	0.142	20.870	1.909
MPI	0.437 **	0.136	10.357	1.549
Situation	0.751 ***	0.136	30.323	2.119
Attitudes	0.817 ***	0.148	30.536	2.263
SN	0.174	0.126	1.900	1.190
Intention	0.897 ***	0.141	40.043	2.452
SA	0.110	0.128	0.744	1.117
Gender				
Male	−0.446	0.271	2.707	0.640
Female	referent			
Age	−0.050	0.149	0.113	0.951
Education				
Primary/Middle school	referent			
High school	−0.046	0.682	0.005	0.955
Undergraduate	0.007	0.588	0.000	1.007
Postgraduate	0.306	0.644	0.226	1.358
Driving license				
With	0.281	0.300	0.882	1.325
Without	referent			
DMPU				
*T* < 2	referent			
2 < *T* ≤ 4	0.372	0.676	0.303	1.451
4 < *T* ≤ 6	0.182	0.690	0.070	1.200
6 < *T*	−0.131	0.712	0.034	0.877

Note: Never using a mobile phone while crossing the street (referent); *** *p* < 0.001, ** *p* < 0.01; B refers to coefficient; S.E. refers to standard error; Wals refer to Wald statistics; Exp(B) refers to odds ratio.

## Data Availability

The data presented in this study are available on request from the corresponding author. The data are not publicly available due to privacy.
